# Development of a Brief Survey on Colon Cancer Screening Knowledge and Attitudes Among Veterans

**Published:** 2005-03-15

**Authors:** Michael S Wolf, Alfred Rademaker, Charles L Bennett, M. Rosario Ferreira, Nancy C Dolan, Marian Fitzgibbon, Terry C Davis, Franklin Medio, Dachao Liu, June Lee

**Affiliations:** Institute for Health Services Research and Policy Studies, Northwestern University, Feinberg School of Medicine; Dr. Wolf is also affiliated with The VA Midwest Center for Health Services and Policy Research, the VA Chicago Healthcare System, Chicago, Ill, and the Robert H. Lurie Comprehensive Cancer Center, Northwestern University, Chicago, Ill; Feinberg School of Medicine; The VA Midwest Center for Health Services and Policy Research, Feinberg School of Medicine, and Robert H. Lurie Comprehensive Cancer Center; The VA Midwest Center for Health Services and Policy Research, Feinberg School of Medicine, and Robert H. Lurie Comprehensive Cancer Center; Feinberg School of Medicine and Robert H. Lurie Comprehensive Cancer Center; Feinberg School of Medicine and Robert H. Lurie Comprehensive Cancer Center; Louisiana State University Medical School, Shreveport, La; Medical University of South Carolina, Charleston, SC; Feinberg School of Medicine; The VA Midwest Center for Health Services and Policy Research

## Abstract

**Introduction:**

Poor knowledge of and negative attitudes toward available screening tests may account in part for colorectal cancer screening rates being the lowest among 17 quality measures reported for the Department of Veterans Affairs health care system, the largest integrated health system in the United States. The purpose of this study was to develop a brief assessment tool to evaluate knowledge and attitudes among veterans toward colorectal cancer screening options.

**Methods:**

A 44-item questionnaire was developed to assess knowledge, attitudes, and beliefs about colorectal cancer and screening and was then administered as part of an ongoing randomized controlled trial among 388 veterans receiving care in a general medicine clinic. Sixteen candidate items on colorectal cancer knowledge, attitudes, and beliefs were selected for further evaluation using principal components analysis. Two sets of items were then further analyzed.

**Results:**

Because the Cronbach α for beliefs was low (α = 0.06), the beliefs subscale was deleted from further consideration. The final scale consisted of seven items: a four-item attitude subscale (α = 0.73) and a three-item knowledge subscale (α = 0.59). Twelve-month follow-up data were used to evaluate predictive validity; improved knowledge and attitudes were significantly associated with completion of flexible sigmoidoscopy (*P* = .004) and completion of either flexible sigmoidoscopy or colonoscopy (*P* = .02).

**Conclusion:**

The two-factor scale offers a parsimonious and reliable measure of colorectal cancer screening knowledge and attitudes among veterans. This colorectal Cancer Screening Survey (CSS) may especially be useful as an evaluative tool in developing and testing of interventions designed to improve screening rates within this population.

## Introduction

Colorectal cancer is the third most common cancer and the third leading cause of cancer death in the United States (1). The U.S. Preventive Services Task Force, the American Cancer Society, and the American Gastroenterological Association have developed guidelines for colorectal cancer screening and recommend that persons aged 50 years or older who are at average risk for the disease be screened periodically (2-4). Despite these recommendations and multiple studies finding that colorectal cancer screening is cost-effective, screening rates are the lowest for any other cancer screening test, with only half of persons aged 50 years and older having received any of the available methods (5).

One potential barrier to effective screening is inadequate knowledge of both the disease and the possible options for undergoing various types of screening tests (6-17). Poor knowledge related to colorectal cancer is associated with compromised perceptions of cancer risk and low rates of screening services use (6,7,12,14,16). Targeted efforts are needed to improve both the overall awareness of colorectal cancer and the availability of often limited resources for invasive screening procedures, such as flexible sigmoidoscopy or colonoscopy. As health education and colorectal cancer screening programs are developed, valid and reliable measures of knowledge and attitude are needed to explicitly assess the efficacy of these efforts.

One prior study reports on a brief instrument that measures beliefs and attitudes toward colorectal cancer screening (17). This assessment was conducted in a mailed survey of primarily white, employed men. However, it has not been evaluated in other settings characterized by higher rates of racial/ethnic minorities or among persons of lower socioeconomic status; these groups have previously been found to be at greater risk for low screening compliance (18-21). Another population at greater risk for low screening compliance is veterans who receive care in the Department of Veterans Affairs (VA) health care system, the largest integrated delivery system in the country. Out of 17 quality measures routinely included in a nationwide VA quality improvement effort, colorectal cancer screening rates are the lowest (22).

As part of a randomized clinical trial effort to improve colorectal cancer screening rates within a VA outpatient general medicine clinic, we recently reported on knowledge and attitudinal barriers to screening participation among veterans (23). Our intervention targeted improvements in both veterans' perceptions about the disease and screening options in addition to their compliance. In this study, we developed and validated a brief measurement tool for evaluating knowledge and attitudes toward colorectal cancer screening among veterans.

## Methods

### Recruitment of participants

We designed a 44-item questionnaire to measure patient knowledge, attitudes, and beliefs associated with colorectal cancer screening and administered the questionnaire to 388 veterans. Male veterans aged 50 years and older who had not received colorectal cancer screening (defined as having a fecal occult blood test [FOBT] within one year or a flexible sigmoidoscopy or colonoscopy within five years) were recruited from general medicine clinics at the VA Chicago Health System between May 1, 2001, and December 31, 2002. Patients were ineligible if they 1) had received a FOBT within one year; 2) received a flexible sigmoidoscopy or colonoscopy within five years; 3) had a personal or family history of colorectal cancer or polyps; or 4) had a personal history of inflammatory bowel disease. In addition, individuals with dementia, impaired vision, hearing problems, or acute illness were deemed ineligible to participate in the study. We excluded patients with impaired vision because the instrument we employed to assess health literacy required the ability to view a list of words. The study protocol was approved by the Northwestern University Institutional Review Board.

Between May 2001 and December 2002, research assistants approached 589 eligible participants as they waited for their scheduled outpatient visit. Of these, 156 (26.4%) refused to be in the study, and 56 (9.6%) did not complete the study questionnaire primarily because their general medicine physician was ready to begin their visit. In all, 388 (65.9%) individuals completed the entire baseline interview, including the questionnaire. No compensation was offered for participation. After the informed consent process, participants took part in a 10- to 15-minute, face-to-face interview that included sociodemographic items, a literacy assessment, and the 44-item questionnaire. The literacy assessment consisted of administering the Rapid Estimate of Adult Literacy in Medicine (REALM), a screening instrument used to determine the ability of patients to read and pronounce common medical terminology and lay terms for body parts and illnesses (24,25). Raw REALM scores are converted to grade ranges: 0–18 = third grade and below, 19–44 = fourth to sixth grade, 45–60 = seventh to eighth grade, and 61–66 = high school. Follow-up interviews were conducted with 227 of these patients six to 12 months after the baseline interview, beginning November 2001 through December 2003. Patients' screening status was obtained through medical record review also during this period.

### Development of the colorectal cancer questionnaire

The 44-item questionnaire included items designed to assess knowledge of colorectal cancer and specific screening tests and attitudes and beliefs toward colorectal cancer and available screening options. Knowledge questions were adapted from the 1992 National Health Interview Survey (NHIS) Cancer Control Supplement, with modifications to reflect current terminology (e.g., use of the term flexible sigmoidoscopy or flex sig instead of proctoscopy) (26). Attitudinal and belief items were developed based on findings from focus groups conducted among this same population of veterans (27). Reader comprehension of the questionnaire items was evaluated using five one-hour cognitive interviews among a convenience sample of community-based, screening-eligible adults. All interviews were conducted by one of the research investigators (Ferreira) and followed available guidelines established for properly conducting cognitive interviews in survey development (28). Interview techniques included both "concurrent think-aloud" and specific probes. The interviews were tape-recorded and abstracted for relevant information, which was used to modify the questionnaire. The modified questionnaire was then administered to a pilot group of 15 patients who were approached in the general medicine clinics (29).

During the pilot process, we obtained patient feedback to items in the pilot test and maintained reading levels of instructions, items, and response options appropriate for lower-literate patients; we used a common measure of document readability (Flesch–Kincaid) to gauge reading levels. Principles described by Doak et al were also applied to maximize item comprehension (30). The final version of the questionnaire registered as having a fifth-grade level of reading comprehension. Even though we planned to administer the instrument through an interview, the readability of the document provided us additional assurance that the questionnaire could be appropriately understood by most patients.

Of the 44 items in the questionnaire administered to the 388 veterans, 10 were associated with knowledge, 29 were associated with attitudes, and five were associated with beliefs. After the administration of the questionnaire, five of the knowledge items were selected by the research team as appropriate for analysis; other knowledge items were excluded because they were conditional questions that were not answered by everyone. Of the 29 attitude items, six were selected for analysis; again, other attitude items were excluded because they were conditional questions not answered by everyone. All five of the belief questions were selected for analysis. Thus, a total of 16 items were selected for analysis.

To prepare for data analysis, we scored questions so that low values reflected high knowledge and attitudes consistent with screening, or "correct" beliefs; high values reflected low knowledge and attitudes inconsistent with screening, or "incorrect" beliefs. Scoring for the knowledge scale was dichotomous (1 = yes, 2 = no); a response of yes required follow-up patient confirmation of understanding of the concept in question. For subjects who responded no or who were determined to have inadequate knowledge of the test in question, simple standard descriptions of both FOBT and flexible sigmoidoscopy were provided by the interviewer to ensure a proper frame of reference. The attitude scale was scored from 1 to 3 on level of worry (1 = not very or not at all worried, 2 = somewhat worried, 3 = very or extremely worried). For both subscales and the total scale, the score was determined by the sum of all nonmissing items. Items on the belief scale were scored for an initial analysis, but the belief construct was not included in a final analysis.

### Psychometric analyses

Principal components (PC) analysis was used to assess the construct validity of the 16 items selected for initial analysis. Cronbach a was used to examine reliability (internal consistency) of the derived knowledge, attitudes, and beliefs subscales. The value of Cronbach a ranges between 0 and 1; if items within a scale are perfectly correlated, then a = 1; if items are totally unrelated, then α = 0. An α coefficient of 0.70 or higher is considered to be acceptably reliable, indicating that items within the same scale measure the same underlying construct. A low Cronbach α for the belief scale and low factor loadings of the belief variables resulted in deletion of this subscale. Final PC analysis on the remaining seven knowledge and attitude items was performed to determine whether these items followed the knowledge and attitude pattern. To confirm reliability of the knowledge and attitudes subscales, correlations between the full scale and items within the subscales were calculated.

An additional question of interest was: Do individuals who improve their knowledge and attitude exhibit different screening behavior than individuals who do not improve knowledge and attitude? To assess the predictive validity of the total score with screening behavior, change in the total score between two time points (initial questionnaire and follow-up questionnaire) was related to screening behavior using Fisher's exact test. It was postulated that an improvement in knowledge and attitudes would be related to an improvement in screening behavior.

## Results

Respondents had a mean age of 67.3 years (SEM = 0.52); 41.4% were African American; 59.6% had completed high school, and 22% had completed college. Respondents' reading abilities averaged at the seventh- to eighth-grade level (mean REALM score = 57.3, SEM = −0.7), with 36% having reading skills lower than the eighth-grade level. More than two thirds (69.1%) of the men in the study were unemployed or retired, and 38% were married. One third of respondents reported their health as either very good or excellent.

Initial analysis consisted of the evaluation of 16 candidate items on colorectal cancer knowledge, attitudes, and beliefs using PC analysis. Item factor loadings for the three-factor solution are shown in Table 1. Because factor loadings for beliefs were low, the belief subscale was deleted from further consideration. Also, decisions were made to remove additional items based on lower factor loadings and/or conceptual fit with remaining items. Thus, the items "likely to get a flexible sigmoidoscopy (or FOBT) if friend recommended," "know testing age," and "heard of colorectal cancer" were deleted from further consideration.

The plan and procedure of item retention resulted in provisional compositions that could be mapped to two factors: knowledge and attitudes. These two sets of items were further analyzed using PC analysis to assess construct validity and Cronbach a to evaluate internal consistency (Table 2). All seven items were retained. The final scale consisted of seven items: a four-item attitude subscale and a three-item knowledge subscale (Table 3).

Higher correlations were observed between items within subscales and their corresponding full scale, while low correlations were expected and subsequently attained between items within subscales and the noncorresponding full scale (Table 4).  

Twelve-month follow-up data were used to evaluate the predictive validity of the knowledge and attitudes scale and each of the two subscales for completion of a colorectal cancer screening test (Table 5). Because low values of all items in the attitudes subscale reflected favorable attitudes consistent with screening, and low values of all items in the knowledge subscale represented high knowledge, decrements over time on these subscales and the overall knowledge and attitude scale represented an improvement in attitudes consistent with screening and/or an improvement in knowledge.

We would assume such improvements in knowledge and attitudes would be associated with screening completion among eligible individuals noncompliant with existing screening recommendations. A minimum decrement over time (i.e., an improvement) of more than four points in the total knowledge and attitude summary scale was significantly associated with higher levels of colorectal cancer screening completion. A decrease of more than four points over time on the full scale was significantly associated with completion of flexible sigmoidoscopy (*P* = .004) and completion of either flexible sigmoidoscopy or colonoscopy (*P* = .02).

## Discussion

We have developed a seven-item scale that can be used to measure knowledge and attitudes toward colorectal cancer screening among U.S. veterans. This instrument (Appendix), the Colorectal Cancer Screening Survey (CSS), was designed to be a brief and simple measure of knowledge and attitudes of veterans toward colorectal cancer screening tests. The results of this study suggest that the two-factor solution offers a parsimonious and reliable measure. It is the first psychometric tool to our knowledge to measure colorectal cancer screening knowledge and attitudes among veterans, a population that is predominantly low-income; nearly half in this study were African American. The CSS was also developed for all levels of literacy. Items were determined to be at a fifth-grade reading level and had simple response options. Moreover, the instrument was interviewer-administered.

Although adequate knowledge and positive attitudes alone may not be sufficient to ensure completion of colorectal cancer screening tests, both are common barriers that have been previously linked to noncompliance. Several studies have found that the absence of clinical symptoms was the most important factor associated with noncompliance with returning FOBTs or undergoing a flexible sigmoidoscopy procedure (7-16). Other attitudinal barriers include fear and anxiety about cancer and perceptions that colorectal cancer screening tests are uncomfortable, embarrassing, or generally unpleasant. The goal of many patient-directed interventions has been to overcome these barriers; the CSS could serve as a valuable indicator of an intervention's efficacy to improve intermediary outcomes.

Interestingly, the CSS had the highest predictive validity with the completion of a flexible sigmoidoscopy screening test, and was less likely to predict screening use when return of FOBTs was considered. This discrepancy may reflect both the level of difficulty of personal endorsement for colorectal cancer screening participation between the available testing options, as well as resources within the VA health care system. For example, the decision to have an FOBT may depend less on knowledge and attitudes than the decision to agree to a more invasive procedure such as flexible sigmoidoscopy or colonoscopy. It may be easier to agree to complete an FOBT with poorer knowledge and a less positive attitude toward colorectal cancer screening than to agree to complete a flexible sigmoidoscopy and colonoscopy, since an FOBT asks less of a patient. Patients may complete the procedure based on physician recommendation without recognizing it as a colorectal cancer screening test. However, flexible sigmoidoscopy and colonoscopy procedures require repeat visits and extensive preparation and take more time to explain and to engage subjects in decision making. Although the relationship did not reach significance, it is noteworthy that those with improved knowledge and attitude scores on the CSS scale had higher rates of colonoscopy screening, a test that is often exceedingly difficult to receive in a timely manner within the VA healthcare system because of limited trained clinical staff and resources.

Limitations to this study should be noted. First, our study is based on a cohort of male veterans. Additional assessments in other settings that provide care for large numbers of racial/ethnic minorities, both male and female, and/or who are of low socioeconomic status, such as the county medical systems, are needed. Second, the CSS may benefit from further psychometric evaluation that could improve upon the knowledge subscale and also evaluate the reliability of CSS scores over time. Further evaluation might also include test–retest reliability and discriminant validity assessments. Evidence of sensitivity to change will be necessary to eventually determine whether the CSS is an applicable evaluative tool for screening interventions.

In conclusion, the CSS may be a useful tool for testing the effect of interventions designed to improve colorectal cancer screening among veterans through improving patient knowledge and attitudes. Because veterans with low knowledge and negative attitudes toward screening tests may not be quickly or easily identified in clinical settings, the CSS might eventually be considered for use as a screening assessment to identify veterans who are at risk for colorectal cancer screening noncompliance.

## Figures and Tables

**Table 1:** Initial Principal Components Analysis of 16 Knowledge, Attitude, and Belief Items, Survey on Colorectal Cancer Screening Among U.S. Veterans^a^

**Factor**	**Factor loading**	**Proportion of variance explained (Eigen value)**	**Cronbach α**

**Attitudes**		0.22 (3.50)	0.63

Likely to get FS if friend recommended	0.69		
Worried FS would be embarrassing	0.71		
Worried FS would be painful	0.69		
Likely to get FOBT if friend recommended	0.65		
Worried FOBT would be embarrassing	0.65		
Worried FOBT would be painful	0.68		

**Beliefs**		0.10 (1.68)	0.06

How serious if found early	−0.04		
Chances of survival if found early	−0.26		
How serious if found late	−0.17		
Chances of survival if found late	−0.12		
Chances of getting colorectal cancer	−0.14		

**Knowledge**		0.10 (1.60)	0.53

Heard of colorectal cancer	0.44		
Heard of tests for colorectal cancer	0.37		
Know of FS	0.22		
Know of FOBT	0.47		
Know testing age	0.31		
**Total percent of variance explained by three factors = 42%.**			

a FS = flexible sigmoidoscopy; FOBT = fecal occult blood test.

**Table 2:** Final Principal Components Analysis of Seven Knowledge and Attitude Items, Survey on Colorectal Cancer Screening Among U.S. Veterans

**Factor**	**Factor loading**	**Proportion of variance explained** **(Eigen value)**	**Cronbach α**

**Attitudes**		0.38 (2.67)	0.73

Worried FS would be embarrassing	0.71		
Worried FS would be painful	0.68		
Worried FOBT would be embarrassing	0.69		
Worried FOBT would be painful	0.73		

**Knowledge**		0.19 (1.33)	0.59

Heard of tests for colorectal cancer	0.50		
Know of FS	0.58		
Know of FOBT	0.53		
**Total percent of variance explained by two factors = 57%.**			

a FS = flexible sigmoidoscopy; FOBT = fecal occult blood test.

**Table 3:** Scores for Participants Responding to Survey on Colorectal Cancer Screening Among U.S. Veterans

** **	**No. Respondents**	**Mean Score**	**SD**	**Range**
7-Item knowledge and attitudes scale	323	9.3	2.2	5-17
3-item knowledge subscale	382	4.7	1.1	2-6
4-item attitude subscale	323	4.7	2.0	2-12

**Table 4:** Item-total Correlations for Scale and Subscales, Survey on Colorectal Cancer Screening Among U.S. Veterans^a^

**Subscales and items**	**Full Scale**	**Subscale**	

		**Attitudes**	**Knowledge**
**Attitude subscale**	0.88	1.00	−0.10
Worried FS would be embarrassing	0.54	0.60	0.02
Worried FS would be painful	0.59	0.63	0.06
Worried FOBT would be embarrassing	0.51	0.53	0.06
Worried FOBT would be painful	0.61	0.64	0.07
**Knowledge subscale**	0.39	−0.10	1.00
Heard of tests for colorectal cancer	0.25	−0.09	0.73
Know of FS	0.18	−0.17	0.69
Know of FOBT	0.33	0.00	0.67

a FS = flexible sigmoidoscopy; FOBT = fecal occult blood test.

**Table 5:** Relationship Between Changes in Scale and Colorectal Cancer Screening Behavior Among U.S. Veterans Participating in Survey and One-year Follow-up Interview (n=227)^a^

**Screening behavior**	**Decrease in Scale >4 points n=69**	**Decrease in Scale ⩽4 points n=158**	***P***
Flexible sigmoidoscopy obtained	16 (23.2)	13 (8.2)	.004
Colonoscopy obtained	13 (18.8)	20 (12.7)	.23
Flexible sigmoidoscopy or colonoscopy obtained	25 (36.2)	33 (20.9)	.02
Flexible sigmoidoscopy, colonoscopy, or FOBT performed	37 (53.6)	87 (55.1)	.89

a Decrease in scale represents improvement in knowledge and attitudes. All values represent numbers (percentages) unless otherwise indicated. FOBT = fecal occult blood test.

## Appendix. The Colorectal Cancer Screening Survey (CSS)

**1. Have you heard of any medical tests to find colon or rectal cancer?**

Yes [1]	No [2]

Colon or rectal cancer is a type of cancer of the large intestine, that is, the part of the body where the stool (or BM or poop) is made; and of the rectum, which is the part of the body the stool (or BM or poop) goes through when you have a bowel movement.

**2. Do you know what a flexible sigmoidoscopy is (also called a “sigmoidoscopy” or “flex sig”)?**

Yes [1] Go to A	No [2] Go to B

A. Can you tell me what it is? *[Open-ended. Check items mentioned. Prompt further explanation without suggestion.]*

- Test done by doctor
- With tube, with light, camera
- The tube is inserted in the rectum
- To look inside for problems/growths/cancer/polyps
- Other (specify) ________________________

B. A flex sig is a test that the doctor does using a flexible tube with a light at the end. The doctor puts the tube in the rectum to check for problems in the rectum or colon. 

*[If respondent is confusing flex sig and colonoscopy]:*

**Colonoscopy:** uses sedation, patient drinks a gallon of bad-tasting liquid to clean out colon.

**Flex sig: **does not use sedation, patient is awake, patient gets an enema to clean out colon.

**3. How worried are you that a flex sig might be embarrassing?  Would you say . . .**

Don’t Know Flex Sig [0]	Not at All Worried [1]	Not Very Worried [2]	Somewhat Worried [3]	Very Worried [4]	Extremely Worried [5]

**4. How worried are you that a flex sig might be painful?  Would you say . . .**

Don’t Know Flex Sig [0]	Not at All Worried [1]	Not Very Worried [2]	Somewhat Worried [3]	Very Worried [4]	Extremely Worried [5]

**5. Do you know what a Fecal Occult Blood Test or Hemoccult Test is (also called an FOBT)?**

Yes [1] Go to A	No [2] Go to B

A. Can you tell me what it is? *[Open-ended. Check items mentioned. Prompt further explanation without suggestion].*

- Collect stool sample at home
- Put it on special cards
- Send to hospital/doctor
- To test if there is blood in the stool
- Other (specify) _____________________________

B. An FOBT is done at home. A person takes a small sample of stool (or BM or poop) and puts it on a special card. Then the card is sent to the hospital and is tested to see if there is blood in the stool (or BM or poop).

*[Make sure respondent is not confusing FOBT with digital rectal exam]:*

**Digital rectal exam:** done by doctor in exam room. Doctor puts stool on special card to test for blood.

**FOBT:** taken home by patient. Patient puts poop onto special card and sends card in to be tested.

**6. How worried are you that an FOBT might be messy?  Would you say . . .**

Don’t Know FOBT [0]	Not at All Worried [1]	Not Very Worried [2]	Somewhat Worried [3]	Very Worried [4]	Extremely Worried [5]

**7. How worried are you that an FOBT might be inconvenient?  Would you say . . .**

Don’t Know FOBT [0]	Not at All Worried [1]	Not Very Worried [2]	Somewhat Worried [3]	Very Worried [4]	Extremely Worried [5]
